# Analysis of Microstrip Line with Asymmetric Arch Type Cross-Sectional Structure Using Micro Pattern Transfer Printing Method

**DOI:** 10.3390/s22155613

**Published:** 2022-07-27

**Authors:** Seungmin Woo, Jaehyeok Choi, Kwangjong Choi, Bokyeong Kang, Hwasun Park, Youngoo Yang

**Affiliations:** 1 Department of Electrical and Computer Engineering, Sungkyunkwan University, 2066 Seobu-ro, Jangan-gu, Suwon 16419, Korea; akrefu@skku.edu (S.W.); tinyh@skku.edu (J.C.); 2 Samwon ACT Inc., 67 Sandan-ro, Danwon-gu, Ansan 15599, Korea; choikj@swact.co.kr (K.C.); kangbk@swact.co.kr (B.K.); 3 Department of Advanced Materials Science and Engineering, Sungkyunkwan University, 2066 Seobu-ro, Jangan-gu, Suwon 16419, Korea; 4 Para-PA Inc., 2066 Seobu-ro, Jangan-gu, Suwon 16419, Korea

**Keywords:** micro pattern transfer printing method, flexible printed circuit board, arch type cross-section, low loss transmission line

## Abstract

This paper presents the manufacturing procedure and electrical properties of a microstrip line on flexible printed circuit boards (FPCBs) fabricated using the micro pattern transfer printing (MPTP) method for millimeter wave band application. The MPTP method presented herein is compared to the conventional FPCB process based on the degree of insertion loss as it pertains to the cross-sectional shape of the formed microstrip line. Electromagnetic field simulations were performed to confirm that the cross-sectional arch shape fabricated by the MPTP process reduces insertion loss in the high-frequency band. Based on the simulation, the microstrip transmission line was optimized to a width of 217 µm and a length of 30 cm, fabricated on a 50 µm thick poly-cyclohexylene dimethylene terephthalate (PCT) substrate to measure the insertion loss. The insertion loss fabricated using the MPTP method is measured as 0.37 dB/cm at 10 GHz, while the conventional FPCB is measured as 0.66 dB/cm. Through the analysis, it was confirmed that the FPCBs manufactured by the MPTP process show lower insertion loss compared to the conventional FPCBs.

## 1. Introduction

There is a growing demand for components of modern electronic devices to exhibit environmental friendliness and cost reduction, as well as high integration, high functionality, and high speed. In particular, such demand is increasing for sensors applied in electric vehicles, such as the Hall sensor. Recently, the industry has taken great interest in reducing the weight and space charge of the circuit boards used for signal transmission of the sensors [1,2,3,4]. Therefore, studies to solve this problem by replacing the regularly printed circuit boards (PCBs) with flexible printed circuit boards (FPCBs) are now drawing attention [5,6,7,8,9]. If the regular PCBs are replaced by FPCBs, fuel efficiency is expected to be improved, reducing the weight of circuit boards connected to the vehicle. In addition, as opposed to the regular PCBs, the FPCBs are made of polyimide or polyester film, so they are thin and flexible; thus, it is possible for them to be bent more freely and reduce their size. Additionally, it has the advantage of being able to withstand high temperatures between 200 and 400 °C compared to other materials [10,11,12]. Due to these characteristics, FPCB has great advantages when it is applied to various sensors. However, the conventional FPCB has limitations in productivity and costs because of the complicated manufacturing processes, such as applying photoresist (PR) to the copper-coated polyimide film, and exposure, development, and drying. This paper proposes a new type of process technology that can replace the conventional process of manufacturing an FPCB sample. The technique proposed in this study is the micro pattern transfer printing (MPTP) method. This is a method of manufacturing FPCB by forming a microcircuit on a metal mold and then transferring it to a film. The advantages of the proposed method are as follows: First, since a metal mold is used, it is possible to reduce the processing steps when manufacturing a circuit. Second, the MPTP method does not require an etching process because it reuses the metal molds in the process instead of etching. Therefore, it reduces the material consumption of chemicals required from the etching process. Third, the MPTP method reduces the surface roughness of the circuit, unlike the conventional method, thereby reducing an insertion loss in the high-frequency band.

In this paper, the MPTP method/manufacturing process and its associated advantages in FPCB fabrication were studied in depth. The differences in the cross-sectional shapes formed during the conventional process were also analyzed against the new MPTP method. The cross-section of the FPCB manufactured through the MPTP process were simulated to analyze the arch shape that reduces the insertion loss at high frequencies. Based on this, a microstrip transmission line with a width of 217 µm and a length of 30 cm was manufactured on a 50 µm thick poly-cyclohexylene dimethylene terephthalate (PCT) substrate. Experimental results of the insertion loss at 10 GHz using an RF signal are presented.

## 2. Analysis

### 2.1. Materials and Methods

**Figure 1:**
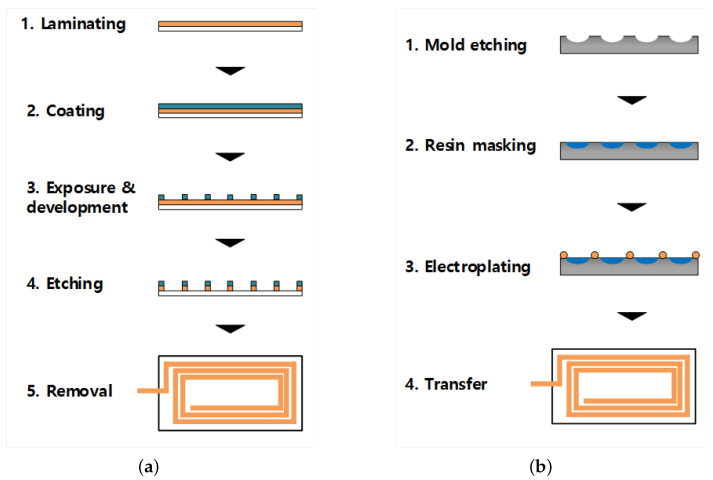
Comparison of fabrication processes: (**a**) The conventional method and (**b**) the MPTP method.

Figure 1a shows the conventional FPCB manufacturing process starting with a structure where the copper foil and polyimide film are laminated. A PR ink is coated onto it and opened through exposure and development. The circuit is formed after etching the copper in the opened area with an etching solution and removing the PR ink. Figure 1b shows that the MPTP process is a technology that forms a circuit pattern on a metal mold and transfers the pattern to a film by electroforming. When the metal mold is electroformed, a copper layer is formed only in the pattern where the metal is exposed, and the copper layer is transferred to an adhesive-coated film. Since the MPTP method uses the manufactured mold again in the process, processes such as exposure, development, and etching are omitted, which is the major difference from the conventional method.

**Figure 2:**
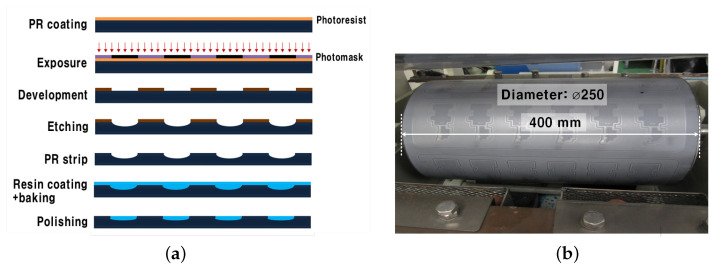
Metal mold information: (**a**) Metal mold fabrication process of the MPTP and (**b**) photograph of the fabricated mold.

Figure 2a shows the metal mold manufacturing process. The first step of the process is to coat a negative PR film on a stainless steel plate. The next step is to project a UV light with a wavelength of 350 nm to form a pattern. After exposure, the pattern is developed using 1% aqueous sodium carbonate solution at 30 °C. Then, the stainless steel substrate is etched using an iron chloride etching solution at 45 °C. After stripping the PR film using 3% aqueous solution of caustic soda at 50 °C, a resin is masked at the etched area, dried/fired for 10–20 s at 80 °C. Finally, the process is completed after polishing the metal mold. Comparing Figure 1a and Figure 2a, the procedures for the fabrication process of the conventional and the MPTP methods are similar. Thus, while the cost and complexity of a single PCM process are similar, the MPTP method reduces the overall cost by reusing the fabricated mold dozens of times.

The etching width and the etching depth are formed through a PCM process after determining the line width. The precision of the metal mold can be improved by an etching process that is optimized during the PCM process. In addition, it is possible to prevent damage to the fine line by improving the electroforming and polishing process. Through the above processes, a metal mold, as shown in Figure 2b, can be manufactured. The manufacturing cost of FPCB using the MPTP method is most affected by the lifespan of the metal mold. This is determined by the adhesion between the polymer resin and the metal mold filled with parts other than the micro pattern. When the plating solution penetrates into the interface between the polymer resin and the metal mold due to multiple plating operations and continuous stress during the transfer process, the polymer resin might be peeled off, hence shortening the lifespan of the metal mold. Therefore, through the development of pretreatment technology that is capable of improving the bonding strength between metal and polymer resin, the lifespan of the metal mold was dramatically enhanced from 30 to more than 100 times.

**Figure 3:**
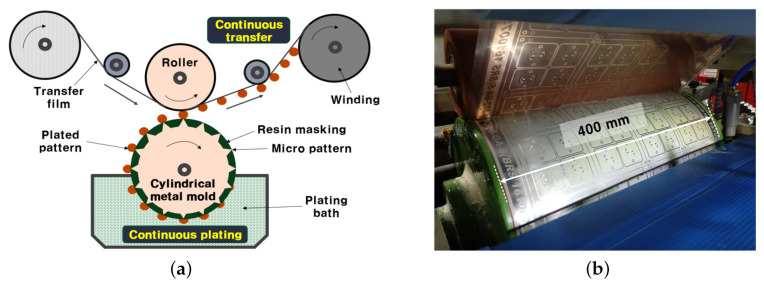
Fabrication procedure using the MPTP method: (**a**) Conceptual diagram and (**b**) photograph of the transfer procedure.

Figure 3 shows the entire process by which a fine pattern is formed in a cylindrical metal mold and transferred to the film multiple times. The conventional fine patterning process requires an expensive and complicated process, such as PR coating/exposure/development/baking/etching as a PCM process on a copper foil film or a resin in the manufacturing process. However, the proposed technology does not use the PCM process every time a fine pattern is formed, and a metal mold with a fine pattern can be manufactured through a single PCM process. The fine pattern is formed on a metal mold by an electroforming process. It is then transferred to a flexible film to which an adhesive is attached. The metal mold whose fine pattern has been formed by the transfer process can be manufactured in large quantities at a low cost by reusing the surface treatment. Since the micro pattern is manufactured by a plating process, the thickness can be easily controlled, and a multi-layer plating and an alloy plating layer can also be fabricated. Therefore, this technology is expected to be used in fields in which it is difficult to fabricate through the conventional PCM processes. Furthermore, since the flexible film can be replaced with materials other than polyimide, it is applicable to various fields.

### 2.2. Simulation-Based Design Optimization

#### 2.2.1. Analysis of the Top Copper Foil Properties

Compared to the conventional method, the MPTP method has two properties that reduce insertion loss. The first is the low surface roughness of the transmission line, and the second is the arch-shaped cross-section. Therefore, in this paper, these two characteristics are analyzed.

**Figure 4:**
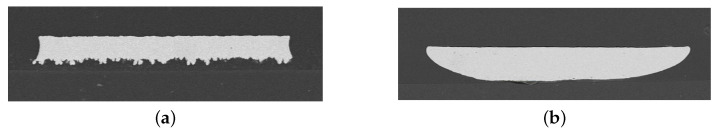
Comparison of the cross-sections formed by each process: (**a**) The conventional process and (**b**) the MPTP process.

The cross-sections of the FPCB fabricated by two different methods were photographed using Philips’ electron microscope, XL 30 ESEM-FEG. Figure 4a is a cross-section of a PCB formed by the conventional process. In the case of the conventional process, roughness has to be generated on the interface between the copper layers and the adhesive layers to increase the adhesion at the surface of the insulation material (polyimide). This characteristic is a factor that increases the insertion loss in the high-frequency band [13,14]. Figure 4b shows a cross-section of the FPCB fabricated by the MPTP process. Through the MPTP method, the circuit is formed in a metal mold and then transferred to the PCB by a compression roller without surface roughness; as shown in Figure 4, the fabricated copper foil becomes smoother in its curvature.

**Figure 5:**
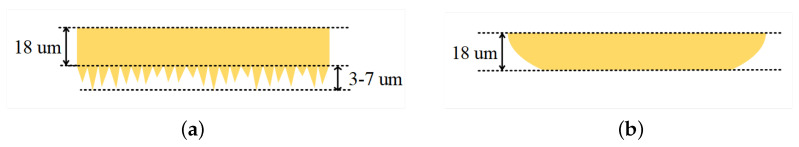
Conceptual diagrams of the cross-section of top copper foil: (**a**) By conventional wet etching and (**b**) by the MPTP method.

Figure 5a shows the surface roughness of the top copper foil. As can be seen, roughness has to be (3–7) µm for the adhesion in the conventional process, and the roughness of the copper foil surface is one of the main causes of more insertion loss in the high-frequency band. In general, the cross-section formed by the conventional method is rectangular with a rugged bottom. Equation (1) shows that the loss of the circuit with such a cross-section is proportional to the surface roughness:

(1) $$\alpha_{C}^{\prime }=\alpha_{C}\left[1+\frac{2}{\pi }\arctan{\left(1.4\frac{\Delta_{0}}{\delta_{s}}\right)}^{2}\right],$$

where $\alpha_{C}$ is the loss of an ideal conductor, $\alpha_{C}^{\prime }$ is the loss of surface roughness, $\delta_{s}$ is the skin depth of the conductors, and Δ is the roughness of the conductor [13,15,16,17].

The electrical properties of the arch-shaped structure at high frequency are described from now on. As can be seen from Figure 4b, the cross-section of the circuit formed by the MPTP process has a unique structure that is different from that of the cross-section formed by the conventional FPCB process. Since the pattern electroplated on the mold is transferred by a compression roller, it has an arch shape. Therefore, it is necessary to analyze the insertion loss through simulation for the arch-shaped cross-section.

**Figure 6:**
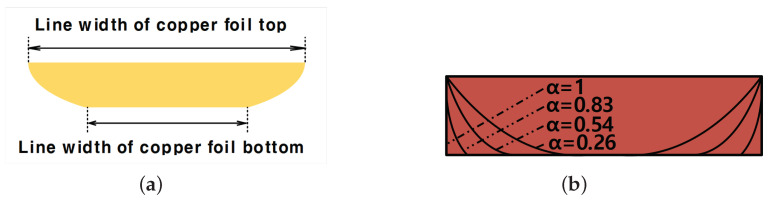
Shape of top copper foil formed due to the MPTP method: (**a**) Schematic of the shape of the top copper foil and (**b**) diagram of the top copper foil according to parameter α.

Figure 6a shows the top copper foil formed by the MPTP method. As mentioned before, in the MPTP method, the top copper has the form of an arch because it is transferred from the metal mold. Parameter α for the ratio to the copper foil top and bottom is defined as follows.

(2) $$\alpha =\frac{line\;width\;of\;copper\;foil\;bottom\;(\mu m)}{line\;width\;of\;copper\;foil\;top\;(\mu m)}$$

**Figure 7:**
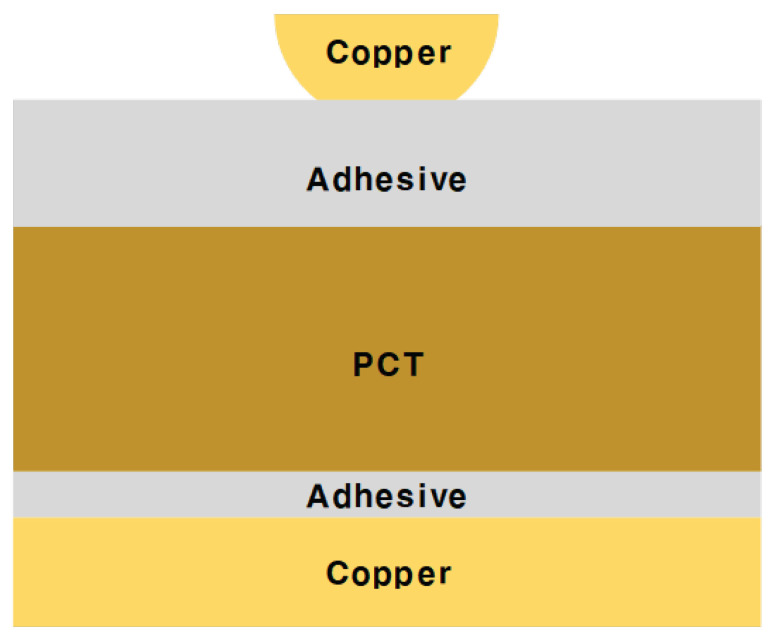
Diagram of the cross-section of FPCB fabricated by the MPTP method.

As shown in Figure 6b, when α becomes 1, the copper foil becomes a rectangle; and as the value of α decreases, the bottom width of the copper foil becomes shorter than the top width. To check the above-mentioned properties through simulation, the cross-sectional layer information of the circuit was configured as shown in Figure 7. Table 1 shows that the dielectric constant and dielectric loss tangent of the adhesive layers were calculated as 2.51 and 0.0226, respectively. The dielectric constant and dielectric loss tangents of PCT were calculated as 2.29 and 0.0068, respectively.

The 3D electro-magnetic (EM) field simulation was conducted for each cross-section using Ansys’ HFSS. The insertion loss was calculated as follows in Equations (3)–(6) based on the simulated 2-port S-parameters [14].

(3) Δ=S(1,1)·S(2,2)−S(2,1)·S(1,2),

(4) $$\zeta =\frac{1-{\left|S\left(1,1\right)\right|}^{2}-{\left|S\left(2,2\right)\right|}^{2}+{\left|\Delta \right|}^{2}}{2|S(1,1)||S(2,2)|},$$

(5) $$G_{max}=\frac{\left|\frac{S(2,1)}{S(1,1)}\right|}{\zeta -{\left(\zeta^{2}-1\right)}^{2}}\cdot 100,$$

(6) $$Insertion\;loss\;(dB)=-10\log\left(\frac{G_{max}}{100}\right).$$

where Δ is the determinant of the scattering matrix, ζ is the stability factor, and $G_{max}$ represents the maximum stable gain. The S-parameters used to calculate the insertion loss were extracted from the EM simulations.

**Figure 8:**
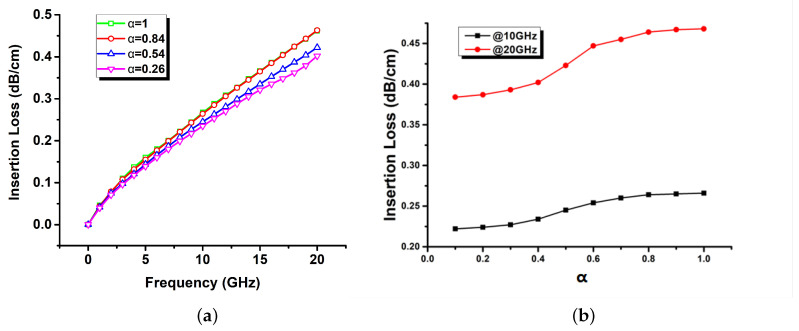
Simulated results of the insertion loss (dB/cm) according to the change of the parameter α: (**a**) Insertion loss according to the frequency and (**b**) insertion loss according to the parameter α.

Figure 8 shows the simulation result of the insertion loss changes according to α. As the value of α decreases from 1, the insertion loss decreases. The form of the copper foil obtained through the etching process is close to α = 1; in contrast, α is reduced when using the MPTP method, so it can be seen that the insertion loss has reduced.

Table 2 shows the result of comparing the values of the graphs of Figure 8 at 3, 10, and 20 GHz. The reduction in insertion loss is proportional to the frequency, and thus it may be seen that when a high-frequency signal propagates on the transmission line, the MPTP method to reduce the parameter α obtains good performance. The reason is that as the α decreases, the bottom surface of the cross-section gets smoother in its curvature.

**Figure 9:**
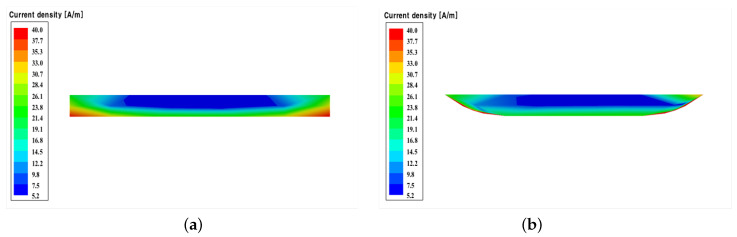
Simulated current density for the cross-sections of the conductors: (**a**) Conductor cross-section using the conventional method and (**b**) conductor cross-section using the MPTP method.

Figure 9a,b show the simulated current densities for the microstrip transmission lines using the conductors having cross-sections from the conventional and MPTP methods at a frequency of 10 GHz. The conductor cross-section for the conventional method has current crowding near the bottom fringes, while that for the MPTP method has more even current distribution through the wider bottom surface of the conductor. This is why the transmission line using the proposed MPTP method has a little lower insertion loss as the value of α decreases.

#### 2.2.2. Optimization of Layer Properties

Figure 7 shows a cross-sectional view of the five layers when fabricating an FPCB using the MPTP method described so far. Flexible PCT was used at the center of the substrate. Adhesive layers were inserted between the PCT and the copper layers. The thickness of a dielectric affects the conductivity and resistance of a circuit. Therefore, a design suitable for the material and structural properties of the PCB substrate used as a substrate of the transmission line is required. In particular, a property is sensitive to thickness since FPCB has a very thin dielectric; thus careful design is required. When using the MPTP method, EM simulation that optimizes the thickness of the adaptive layer and the PCT is required. Therefore, the tendency is checked while simulating the PCT and adhesive to have a minimized insertion loss within the range that can be implemented.

**Figure 10:**
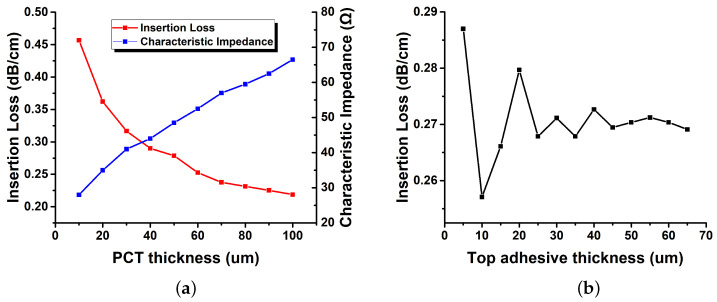
Simulated results of the variation of layers: (**a**) Variation of PCT thickness and (**b**) variation of top adhesive.

The red line in Figure 10a is the simulation result of the insertion loss of the transmission line as the PCT thickness increases. As the thickness of the PCT increases, the insertion loss decreases significantly, and then the decreasing trend is gradually mitigated from 50 µm or more. Therefore, the PCT was designed with 50 µm as the maximum thickness that can be implemented. The blue line is the simulation result of how the characteristic impedance changes for constant line width when the PCT thickness is increased. When the line width is fixed at 217 µm, the characteristic impedance increases as the PCT thickness increases. This is the same as the effect of decreasing the dielectric constant when the thickness of the dielectric is fixed. That is, to minimize the line width, the thickness of the PCT has to be minimized too. If the characteristic impedance of the transmission line is fixed at 50 Ω, the width and insertion loss have a trade-off relationship. Therefore, the design should be appropriately selected in consideration of the performance properties to be simultaneously satisfied. In this paper, we decided the thickness of PCT to be 50 µm with a minimum loss while satisfying a line width of 217 µm. The solid line in Figure 10b is the simulation result of the insertion loss of the transmission line as the thickness of the top adhesive layer increases. When the thickness is low, the insertion loss changes in response to the change in thickness. However, due to the process implementation capability, if the adhesive thickness is reduced when implementing the adhesive thickness, there is a deviation. Therefore, to minimize the deviation between samples, it would be appropriate to use a top-adhesive layer of 25 µm or more, considering the process’s implementation capability.

**Figure 11:**
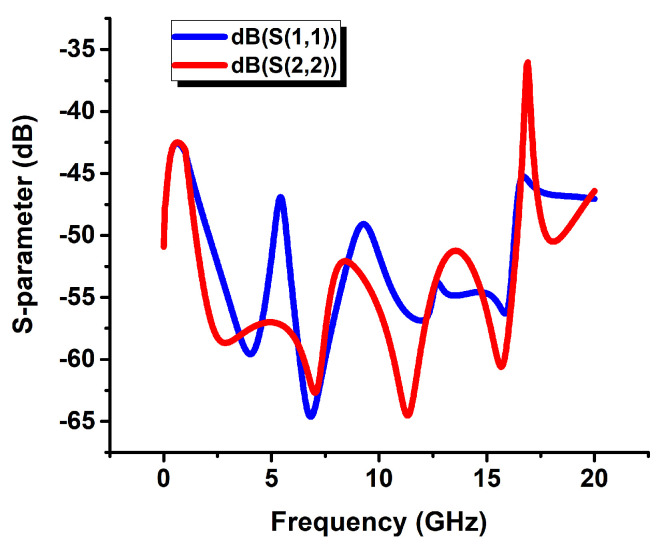
Simulated 50 Ω impedance matching results of the transmission line.

The transmission line was matched to 50 Ω. Figure 11 shows the simulation results for 50 Ω impedance matching. The S-parameter values, which are S(1,1) and S(2,2) values in the dB scale, are below −40 dB in the (0–20) GHz frequency band when the width of the line is 217 µm, so it is checked that the line is properly matched to 50 Ω.

The second column of Table 3 shows the thickness of each layer for the conventional method. The last column of Table 3 shows the thickness of each layer for the MPTP method that has been optimized according to the EM simulation results. As shown in the table, it was determined that the top and bottom copper were 18 µm and the PCT be 50 µm for both cases of the conventional method and the MPTP method. In the case of the MPTP method, adhesive layers are required, unlike the conventional method. The top adhesive and the bottom adhesive were 25 and 5 µm, respectively, whereas the total thickness was set at 116 µm.

## 3. Implementation and Experimental Results

**Figure 12:**
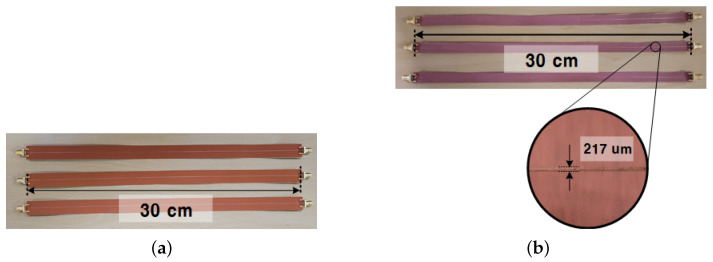
Photograph of the fabricated FPCB samples with two different methods: (**a**) Fabricated samples using the conventional method and (**b**) using the MPTP method.

**Figure 13:**
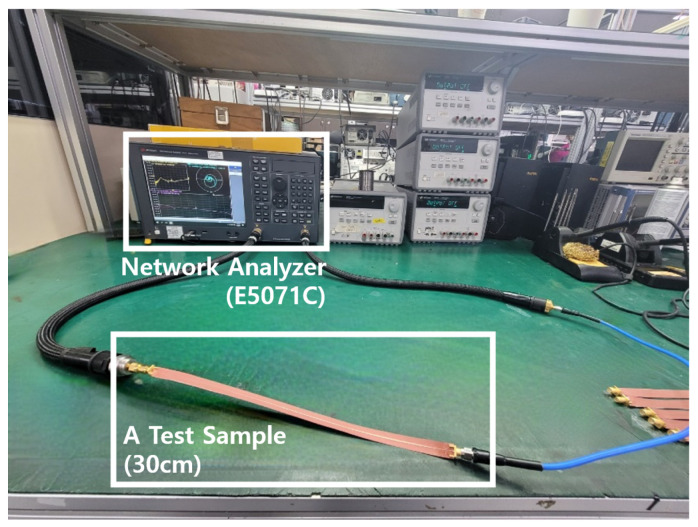
Photograph of the test bench using the network analyzer (E5071C).

Figure 12 shows the fabricated FPCB samples using two different methods: The conventional method and the MPTP method. For connection with RF cables, SMA connectors are attached to both sides of the transmission line of the sample. Figure 13 shows the test bench for fabricated samples. S-parameters of the samples were measured from 0 to 20 GHz using a network analyzer (E5071C) by Keysight.

**Figure 14:**
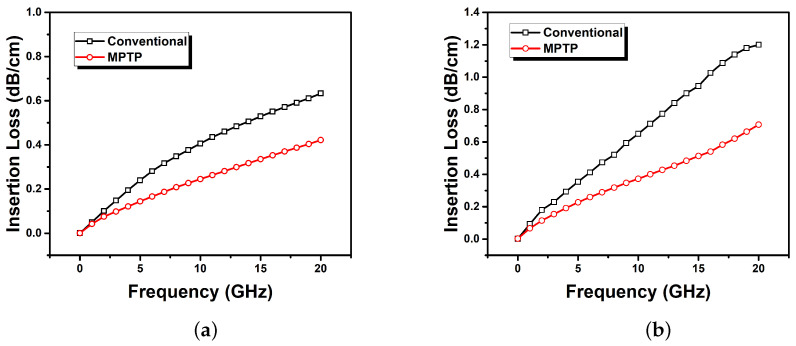
The insertion loss of the FPCB samples: (**a**) Simulation and (**b**) measurement.

Figure 14 shows the simulated and measured insertion loss per cm for the fabricated samples using the conventional and MPTP methods. From the measurements, the insertion losses of 0.66 and 0.37 dB/cm were obtained at a frequency of 10 GHz for the samples fabricated using the conventional and MPTP methods, respectively. Therefore, the insertion loss is improved by 0.28 dB/cm using the MPTP method compared to the conventional method. For both the conventional and MPTP methods, the difference between the measurement and simulation may come from the loss tangent value used in simulation, which was measured only at 2 GHz due to the limited capability of the measurement instruments.

## 4. Conclusions

In this paper, the manufacturing process and the properties of the FPCB circuit fabricated using the MPTP method were studied, compared to the conventional method. The major difference between the MPTP method and the conventional method in the manufacturing process is that the MPTP method reuses the metal mold in the process, so an etching step is not required after the metal mold is manufactured. With respect to the property, differences between a cross-section formed by the conventional process and the MPTP process have been analyzed. The simulation results confirmed that the arch-shape manufactured by the MPTP process reduces the insertion loss proportional to the frequency. The simulation showed that as the width of the bottom surface of the cross-section becomes narrower than the top surface, the insertion loss decreases, and thus, it is confirmed to decrease by a maximum of 0.03 dB/cm at 10 GHz. In addition, the cross-section of the circuit manufactured by the MPTP method has a reduced surface roughness compared to the circuit formed by the conventional method, which results in insertion loss improvement, especially at high frequencies. The measurement results verified that the insertion loss could be reduced using the MPTP method. However, as a result of the experiment, a loss difference occurred in the insertion loss between the simulation and measurement in both cases of the conventional method and the MPTP method. In the future, we will conduct additional research to minimize these differences.

## Figures and Tables

**Table 1:** Properties of the PCT and adhesive layers.

Layer	Dielectric Constant	Dielectric Loss Tangent
Adhesive	2.51	0.0226
PCT	2.29	0.0068

**Table 2:** Insertion loss (dB/cm) according to parameter α.

α	3 GHz	10 GHz	20 GHz
1	0.109	0.266	0.462
0.84	0.108	0.264	0.462
0.54	0.098	0.245	0.421
0.26	0.095	0.235	0.401

**Table 3:** The optimized thickness of each layer.

Layer	Conventional Method	MPTP Method
	Thickness (µm)	Thickness (µm)
Top copper	18	18
Top adhesive	-	25
PCT	50	50
Bottom adhesive	-	5
Bottom copper	18	18

## Data Availability

Not applicable.
